# Compliance as a stable function in the treatment course of bipolar disorder in patients stabilized on olanzapine: results from a 24-month observational study

**DOI:** 10.1186/s40345-014-0013-x

**Published:** 2014-10-23

**Authors:** Alexandra Kutzelnigg, Martin Kopeinig, Chih-Ken Chen, Ágnes Fábián, María Gloria Pujol-Luna, Young-chul Shin, Tamás Treuer, Yulia D’yachkova, Claudia Deix, Siegfried Kasper, Dagmar Doby

**Affiliations:** Department of Psychiatry and Psychotherapy, Division of Biological Psychiatry, Medical University Vienna, Waehringer Guertel 18-20, 1090 Vienna, Austria; Psychosoziale Dienste in Wien (PSD), Mariahilfer Strasse 77-79, 1060 Vienna, Austria; Chang Gung Memorial Hospital, Keelung, Chang Gung University School of Medicine, No.200 Lane 208, Ji-Jin 1st Road, Anle District, Keelung City, 204 Taoyuan, Taiwan; Réthy Pál Kórház-Rendelőintézet, Békéscsaba, Hungary; Hosp. Angeles Metropolitano, 59-600 Roma Sur, 06760 Mexico City, Mexico; Kangbuk Samsung Hospital, 110-746, Seoul, South Korea; Eli Lilly, Madách u. 13-14. (VII. emelet), 1075 Budapest, Hungary; Eli Lilly, Kölblgasse 8-10, 1030 Vienna, Austria

**Keywords:** Bipolar disorder, Compliance, Long-term treatment, Quality of life, Drug attitude, Olanzapine

## Abstract

Compliance is a key factor in the maintenance treatment of bipolar disorder. This noninterventional study was conducted to explore factors associated with higher levels of compliance in bipolar patients, all treated in routine clinical settings. Bipolar outpatients (Clinical Global Impression of Severity score ≤3) who had been stabilized with olanzapine mono- or combination therapy for ≥4 weeks were enrolled in the study. Compliance to medication was assessed at baseline and after 3, 6, 9, 12, 18, and 24 months by a physician-rated, 4-point categorical scale using the following classification: noncompliant (patients being compliant to treatment schedule less than 20% of the time) and low (20% to 59% of the time), moderate (60% to 79% of the time), and high (≥80% of the time) levels of compliance. Both baseline and post-baseline factors were used in a generalized estimating equations (GEE) model to predict the likelihood of high compliance. Of 891 eligible patients, 657 patients completed the 24-month observation period. High levels of compliance (≥80%) were observed in 67% of patients at baseline, increasing to 80% in study completers. High compliance at baseline was identified as a strong predictor of compliance during study participation (odds ratio (OR) = 6.9, 95% confidence interval (CI) = 5.0 to 9.5, *p* < 0.001). Factors associated with high compliance during the study (GEE model) included greater life satisfaction (*p* = 0.002), better insight into illness (*p* < 0.001), less work impairment (*p* = 0.007), and fewer days of inpatient care (*p* = 0.002). Compliance ratings varied by country (*p* < 0.001) and duration of post-baseline treatment (*p* = 0.014). In conclusion, a number of clinical, functional, and social factors were identified as predictors of compliance in patients with bipolar disorder. As compliance is crucial for the long-term management of these patients, more attention should be directed towards compliance itself and factors associated with compliance levels in everyday treatment settings.

## Background

Compliance with pharmacological treatment is crucial for response to medication and for long-term outcome in any chronic medical condition (Haynes et al. 2002). Compliance is usually higher in patients with acute illness compared to patients with chronic disease, dropping substantially after the first 6 months of treatment (Cramer et al. 2003; Jackevicius et al. 2002). Despite the great potential to benefit from stable and long-term treatment regimens, patients with chronic mental illness may frequently face more and specific difficulties in being compliant compared to nonpsychiatric patients, mainly due to a lack of insight, cognitive deficits, and (dis)beliefs regarding the efficacy and safety profiles of medication (Cramer and Rosenheck 1998; Osterberg and Blaschke 2005; Pompili et al. 2013; Zygmunt et al. 2002). Noncompliance is usually difficult for clinicians to detect and is thus underestimated in routine clinical settings. However, it is strongly related to relevant clinical outcomes such as relapse, (re)hospitalization, and suicide attempts (Novick et al. 2010), especially in schizophrenia and bipolar disorder (Velligan et al. 2009). Furthermore, noncompliance in patients with severe psychiatric illness may have a substantial economic impact (Murray and Lopez 1996; Sun et al. 2007)*.*

Bipolar disorder is a common and often severe mental illness with a lifetime prevalence of 1% to 3% (Judd and Akiskal 2003; Regeer et al. 2004), which is associated with a high risk for relapse (Li et al. 2014; Sharma et al. 2014; Simhandl et al. 2014). Noncompliance with medication has been frequently reported for bipolar patients, with a reported incidence ranging from 20% to 60% (Adams and Scott 2000; Colom and Vieta 2004; Gonzalez-Pinto et al. 2006). Several pharmacological treatment strategies have been proposed for relapse prevention in patients with bipolar disorder (Beynon et al. 2009) such as mood stabilizers (Goodwin et al. 2003; Grunze et al. 2009) and, according to more recent guidelines, second-generation antipsychotics (Goodwin 2009; Grunze et al. 2009, 2010; Yatham et al. 2013; Hirschfield 2014). Olanzapine is considered as first-line treatment for bipolar I disorder for both acute episodes and relapse prevention (Grunze et al. 2009; Yatham et al. 2006) and has been systematically studied in a large number of studies as monotherapy or in combination with mood stabilizers and antidepressants, such as fluoxetine, in all of these indications (Baker et al. 2003; Tohen et al. 1999, 2002, 2003, 2005, 2006).

Although the efficacy, safety, and tolerability of psychopharmacological agents used to treat bipolar patients have been widely evaluated (Beynon et al. 2009), the current study is the first to evaluate compliance as the primary outcome in long-term, routine treatment settings and within a large patient population. The aims of the study were to determine factors associated with better compliance in patients with bipolar disorder stabilized on olanzapine and to assess whether there was a difference in compliance between patients stabilized on olanzapine monotherapy and those stabilized on combination therapy. The assessment of compliance was prioritized in our study, as it might be one important factor contributing to the lack of generalizability of clinical trial data and can be influenced in routine clinical care by a variety of intervention strategies.

## Methods

### Study design

This prospective, noninterventional, 24-month observational study (study code F1D-OE-B015) was conducted to address the need for further information regarding compliance in patients receiving long-term treatment for bipolar disorder. Stable bipolar outpatients were recruited from January 2005 to December 2006 in six countries (Austria, Romania, Hungary, Korea, Taiwan, and Mexico). The study protocol was acknowledged or approved by the relevant ethics committees. All patients and/or their authorized legal representatives provided written informed consent before screening. Treatment was prescribed in a standard-of-care setting at the discretion of the treating psychiatrist and was not provided by the sponsor during or after participation in the study. Treatment changes, as well as discontinuation of medication at any time after baseline, were permitted and did not lead to discontinuation of the respective patients. Concomitant medication at any point in time and for any duration of treatment during study participation was also allowed in order to reflect routine clinical settings.

### Patients

To maximize generalizability and in line with the observational design of the study, minimal patient eligibility criteria were applied. Patients were enrolled at the discretion of their psychiatrist if all four conditions were satisfied: (1) presented within the standard course of care and were seen in an outpatient setting for the long-term treatment of bipolar disorder, (2) had received olanzapine oral medication alone or in combination with a mood stabilizer for the treatment of bipolar disorder for at least 4 weeks before study entry and were in stable psychiatric condition at screening (Clinical Global Impression of Severity (CGI-S) score (Guy 1976) ≤3), (3) were at least 18 years of age, and (4) were not currently participating in any other clinical trial with an interventional design. Data were collected during the normal course of patient care at the baseline visit and at routine follow-up visits, which took place at approximately 3, 6, 9, 12, 18, and 24 months thereafter.

### Measures

As the study was designed to reflect everyday clinical treatment settings, only measures, which are also commonly used in routine clinical care, were administered. No specific diagnostic instruments were used to screen for bipolar disorder, but physicians were asked to establish the diagnosis according to their routine clinical approach. Patients were further diagnosed as having bipolar I or bipolar II disorder, as suffering from rapid cycling or not, and as having a current manic/hypomanic or depressive episode. The presence or absence of psychotic symptoms during the current episode was also assessed. Besides differential diagnosis, data on sex, age, race, education status, psychiatric and treatment history (including record of suicide attempts), and height were collected at baseline to characterize the patient sample and detect initial differences between groups. The following variables were collected at baseline and at each study follow-up visit: (1) treatment regimen(s), duration of treatment, and level of compliance, (2) patient attitude to medication, (3) disease severity, (4) medical resource use, (5) functional status, (6) relapse status, (7) patient-physician relationship, (8) quality of life, (9) comorbidities and concomitant medication, and (10) weight. These variables were assessed as follows.

(1) The main outcome measure was compliance with the prescribed treatment regimen. This was evaluated subjectively by the treating psychiatrist by assigning the patients' compliance level to one of the four following categories: high (patients being compliant with the prescribed medication, appointments, and all other interventions 80% to 100% of the time), moderate (patients being compliant with all aspects of treatment 60% to 79% of the time), low (patients being compliant 20% to 59% of the time), and noncompliant (patients being compliant <20% of the time). (2) In addition, the Drug Attitude Inventory - short version (DAI-10) (Awad 1993) was used to measure patients' attitude towards medication. Higher scores on the scale denote more affirmative attitudes towards medication. (3) Disease severity was measured by the CGI-S. (4) Information on medical resource use related to bipolar disorder was captured in a short questionnaire by quantifying the number of outpatient consultations, number of inpatient admissions, number of days spent in an inpatient facility, and number of days on sick leave. (5) Functional status was assessed by the physicians using a short categorical questionnaire to capture the number of social activities (with categories from 0 to ≥5), level of current work activity (categories: unable to work, unemployed, retired, student, housekeeping, sheltered program, volunteer work, working for pay), impairment in work activities (categories: unable to work due to mental illness, severe, moderate, or no impairment), and satisfaction with life (categories: very dissatisfied, dissatisfied, neither satisfied nor dissatisfied, satisfied, very satisfied). (6) Relapse status was determined as per the investigator's clinical assessment (based on exacerbation of acute symptomatology, hospitalization, etc.) and captured directly on the data collection form using questions with a simple ‘yes’ or ‘no’ answer and in case of a relapse the date when relapse was first noticed. (7) A 5-point categorical scale was used to record the strength of the patient-physician relationship (categories: poor, fair, good, very good, excellent), as well as the patient's insight into illness (categories: none, low, medium, moderate, high). (8) Patient quality of life was assessed by the European Quality of Life instrument-5 dimensions (EQ-5D) (The EuroQol Group 1990), which consists of a patient-rated five-dimension questionnaire and a visual analog scale (VAS). (9) Comorbidities, use of concomitant medication, and tolerability (expressed as the presence of side effects judged to be associated with the patient's condition/treatment) during the 4 weeks before baseline and since the last visit, respectively, were captured.

### Statistical methods

The sample size calculation was based on the assumptions of 80% completion rates, 30% of patients receiving monotherapy, 70% receiving polytherapy, and corresponding compliance rates of 73% and 52%, respectively (Greenberg 1984). The precision of the estimate for the difference in compliance between mono- and polytherapy treatment groups was used as the basis for the sample size calculation. Based on the above assumptions and a sample of 960 patients, the 95% confidence interval (CI) width for a difference in such proportions is ±7.1. Missing data were not imputed; all eligible patients were used for analyses. Due to the observational design, primarily descriptive statistics were used: means, standard deviations, and frequencies.

For the primary analysis of factors associated with compliance, the categories of compliance (high, moderate, low, and noncompliant) were dichotomized into ‘high’ compliance (≥80% compliance, corresponding to the high compliance category) and ‘low’ compliance (<80% compliance, a pooled group comprised of the moderate, low, and noncompliance categories). This classification was made as a result of the rare occurrence of low and noncompliance ratings (see the Results section). Baseline characteristics were compared between compliance levels by the chi-square test for categorical variables and by the *t*-test for continuous variables. Time to relapse was plotted by the Kaplan-Meier approach. The generalized estimating equations (GEE) approach was used to model evaluations of compliance collected at multiple visits during the study as a function of various baseline and post-baseline (i.e., time-varying) covariates. Some patients were prescribed a number of different drug regimens during the 3- or 6-month intervals between study visits and therefore had more than one compliance rating recorded at the corresponding visits. In case of such regimen changes between study visits, the lowest recorded compliance level was used for analyses.

Patient characteristics were analyzed at each visit when olanzapine-containing regimens were recorded. Due to the low proportion of patients not receiving olanzapine (proportions ranged from 2% to 11% between visits), they were not summarized separately. Due to the high number of baseline covariates collected in the study, only variables different at baseline at a 10% significance level and variables thought to be influential (country effect, treatment as mono- versus polytherapy) were entered into the initial joint model for compliance and were then reduced using stepwise procedures. At the next step, post-baseline values for baseline variables selected by the stepwise procedure were added to the model, and model reduction using stepwise selection was applied.

Secondary analyses included similar GEE modeling with repeated measures of total DAI-10 score over time and quality of life over time (total EQ-5D score derived from five domains and EQ-5D VAS score) as a function of the independent variables suggested by clinicians: country, education, olanzapine mono- versus polytherapy, interaction of treatment regimen by treatment duration, overall CGI-S score, patient-physician relationship, patient insight into their disease, indication of treatment for other psychiatric disorders, total EQ-5D score, EQ-5D VAS score, compliance level, age, and gender. Similar models were used to evaluate quality of life over time, measured by total EQ-5D score derived from five domains and by EQ-5D VAS score. Similar repeated measures logistic regression modeling of compliance (GEE) was performed with the ten sub-scores of the DAI-10 used as predictors. Two analyses were performed to study the association of compliance with relapse: logistic regression modeling to look at the presence/absence of relapse and Cox regression analysis to model the time to relapse. Because of the significant differences in health-care systems of the participating countries and different derivation of the costs used in the study (psychiatric hospitalization (with an overnight stay), outpatient consultations with a psychiatrist, and day hospital or day care facility use), UK 2007/2008 estimates of these costs were used in the analyses. The relationship between total medical resource utilization and total compliance over 2 years was modeled using linear regression for total cost based on UK estimates (also see the Results section) by baseline and post-baseline compliance levels adjusted for significant baseline variables, country, and treatment (mono/polytherapy and duration). Since compliance was of primary interest in the model, it was retained regardless of the level of significance.

## Results

### Patient disposition

**Figure 1:**
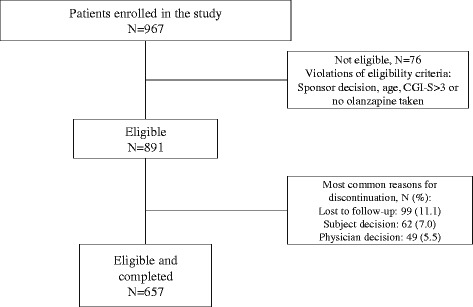
**Patient disposition.** CGI-S, Clinical Global Impression of Severity.

As presented in the study flow diagram (Figure 1), 967 patients were enrolled in the study. Of these, 891 patients were eligible to participate: 239 (26.8%) in Austria, 180 (20.2%) in Romania, 167 (18.7%) in Hungary, 145 (16.3%) in Korea, 99 (11.1%) in Taiwan, and 61 (6.8%) in Mexico. The most common reasons for noneligibility were sponsor decision, age <18 years, CGI-S score >3, or no olanzapine treatment before screening. A total of 657 patients (73.7% of eligible patients) completed the 24-month study; the most common reasons for discontinuation from the study were lost to follow-up (11.1%), subject decision (7.0%), and physician decision (5.5%).

### Compliance level at baseline

In all countries, most patients were rated as being ‘highly compliant,’ followed by ‘moderately compliant’; low and noncompliant ratings constituted a very low proportion at baseline. As presented in Table 1, the percentages of patients receiving the different compliance level ratings were not evenly distributed in the participating countries, with the greatest proportion of high compliance ratings found in Austria and in Korea and the lowest proportion in Romania and in Taiwan. Low compliance ratings were most frequently reported in Taiwan; the highest proportion of noncompliance was recorded in Mexico.

**Table 1:** **Compliance level at baseline**

	**Eligible at baseline**	**High (≥80%)**	**Moderate (60% to 79%)**	**Low (20% to 59%)**	**Noncompliant (<20%)**
Austria	239 (26.8)	187 (78.2)	43 (18.0)	7 (2.9)	2 (0.8)
Romania	180 (20.2)	89 (49.4)	78 (43.3)	11 (6.1)	2 (1.1)
Hungary	167 (18.7)	112 (67.1)	51 (30.5)	4 (2.4)	0
Korea	145 (16.3)	110 (75.9)	27 (18.6)	7 (4.8)	1 (0.7)
Taiwan	99 (11.1)	59 (59.6)	24 (24.2)	12 (12.1)	4 (4.0)
Mexico	61 (6.8)	37 (60.7)	18 (29.5)	3 (4.9)	3 (4.9)
Total	891 (100)	594 (66.7)	241 (27.0)	44 (4.9)	12 (1.3)

All data presented as *n* (%).

Table 1 also shows the proportion of patients with each compliance rating at baseline. The ‘high compliance group’ (HCG; ≥80% compliance) comprised 594 patients (66.7%), while the ‘low compliance group’ (LCG; <80% compliance) comprised 297 patients (33.3%).

### Baseline characteristics by level of compliance

Baseline characteristics are summarized in Table 2. There were no significant differences regarding age, weight, height, gender, and race between the HCG and LCG; however, regarding functional and clinical characteristics, the following significant differences were seen: compared to patients in the LCG, patients in the HCG had a lower mean CGI-S score, had a higher mean DAI-10 score, had spent less time in hospital due to bipolar disorder in the year before entering the study, had fewer relapses in the last 4 weeks before study entry, had a higher mean EQ-5D overall health status score, and had a higher mean EQ-5D VAS score at baseline. Patients in the HCG also had significantly more often participated in social activities with friends or social groups, had higher level of work activities, had less impairment in work activities, had a better patient-physician relationship, had greater insight into their illness, and had remained longer on an unchanged treatment regimen before baseline. Psychiatric comorbidities were reported very infrequently at baseline and also throughout the study. Seventy-one percent of patients in the HCG and 65% in the LCG (*p* = 0.065) mentioned no comorbidity at all in the 4 weeks before baseline. At the beginning of the study, 44.6% of patients were treated with olanzapine monotherapy, while 55.4% received olanzapine combination therapy (of those, 87.8% in combination with a mood stabilizer).

**Table 2:** **Patient baseline characteristics by level of compliance**

	**High (≥80%)**	**Low (<80%)**	***p*** **value**
Demographics			
Age, years, mean (SD)	43.0 (12.8)	43.8 (12.9)	0.44
Weight, kg, mean (SD)	73.5 (13.8)	73.9 (14.1)	0.69
Height, cm, mean (SD)	168.1 (9.3)	167.1 (8.8)	0.11
Sex, females, *n* (%)	337 (56.7)	176 (59.3)	0.47
Race, *n* (%)			0.30
Caucasian	384 (65.1)	198 (67.3)	
Hispanic	37 (6.3)	24 (8.2)	
East Asian	169 (28.6)	72 (24.5)	
Clinical characteristics			
CGI-S, mean (SD)	2.4 (0.7)	2.6 (0.7)	0.002
DAI-10, mean (SD)	5.7 (3.7)	4.8 (4.0)	0.002
Days bipolar inpatient last year, mean (SD)	15.4 (38.5)	18.4 (32.8)	0.029
Recent relapse, *n* (%)	68 (11.4)	68 (22.9)	<0.001
Functional characteristics			
EQ-5D overall health status score (SD)	0.9 (0.2)	0.8 (0.2)	0.0012
EQ-5D VAS score (SD)	74.2 (15.4)	69.6 (17.6)	<0.001
Number of activities with friends or social groups, *n* (%)			
0	100 (16.8)	85 (29.0)	
1	66 (11.1)	40 (13.7)	<0.001
2	117 (19.7)	49 (16.7)	
3	78 (13.1)	40 (13.7)	
4	70 (11.8)	20 (6.8)	
≥5	163 (27.4)	59 (20.1)	
Work activity, *n* (%)			
Unable to work	34 (5.8)	26 (9.0)	<0.001
Volunteer work	21 (3.6)	6 (2.1)	
Student	34 (5.8)	12 (4.1)	
Working for pay	199 (33.8)	76 (26.2)	
Keeping house	109 (18.5)	48 (16.6)	
Unemployed	59 (10.0)	23 (7.9)	
Sheltered program	15 (2.5)	3 (1.0)	
Retired	118 (20.0)	96 (33.1)	
Impairment in work activities, *n* (%)			
No impairment	123 (20.8)	39 (13.3)	
Mild impairment	220 (37.2)	81 (27.6)	
Moderate impairment	150 (25.4)	133 (45.2)	
Severe impairment	50 (8.5)	22 (7.5)	
Unable to work due to mental illness	31 (5.2)	15 (5.1)	<0.001
Patient-physician relationship, *n* (%)			
Poor	0	7 (2.4)	<0.001
Fair	20 (3.4)	25 (8.4)	
Good	158 (26.6)	148 (49.8)	
Very good	277 (46.6)	101 (34.0)	
Excellent	139 (23.4)	16 (5.4)	
Insight into illness, *n* (%)			
None	3 (0.5)	3 (1.0)	
Low	26 (4.4)	49 (16.5)	<0.001
Medium	87 (14.6)	111 (37.4)	
Moderate	168 (28.3)	93 (31.3)	
High	310 (52.2)	41 (13.8)	
Length of previous treatment regimen (4 weeks before baseline, containing olanzapine), *n* (%)			
<14 days	8 (1.3)	10 (3.5)	
15 to 30 days	125 (21.1)	91 (31.5)	<0.001
31 to 90 days	212 (35.8)	77 (26.6)	
>90 days	244 (41.1)	101 (34.9)	

CGI-S, Clinical Global Impression of Severity; DAI-10, Drug Attitude Inventory - short version; EQ-5D, European Quality of Life instrument-5 dimensions; SD, standard deviation; VAS, visual analog scale.

### Compliance and factors associated with compliance during the study

**Figure 2:**
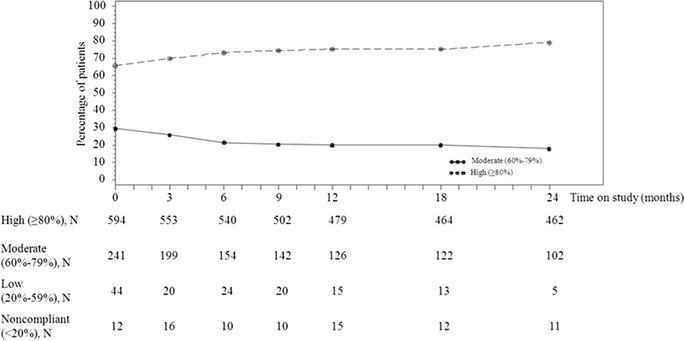
**Compliance during study (number and percentage of patients on olanzapine-containing regimens).** ‘Compliance’ and ‘noncompliant’ patients were too low to be shown.

Figure 2 shows the proportion of patients rated as being ‘highly’ and ‘moderately’ compliant at each visit during the study (the proportion of patients showing low compliance and noncompliance accounts for the remaining percentages and is too low to be shown in the graph). The proportion of patients rated as being ‘highly’ compliant increased continuously from one visit to the next, from 67% at baseline to almost 80% at the last visit; conversely, the proportion of patients rated as being ‘moderately’ compliant showed a steady decrease from 27% to 18%. This shift could be associated with a higher discontinuation rate in patients with lower compliance. No significant difference in compliance was observed between patients taking olanzapine monotherapy and those taking olanzapine in combination with other drugs.

Table 3 presents factors that were significantly associated with compliance during the study. Compliance during the study (modeled as a trend over time) was strongly related to the baseline compliance rating, with patients who had a high score at baseline having almost sevenfold higher odds of being rated as ‘highly’ compliant during the study. There was a strong variation in ratings between countries, with patients in Mexico having four times higher odds of receiving ‘high’ ratings than patients in Taiwan. Better insight into illness was strongly associated with higher levels of compliance, with an almost threefold increase in the odds of high compliance from moderate to high insight and a threefold decrease from moderate to low/none. We found a nonlinear relationship between the duration of the first treatment regimen and compliance, in that the rate of compliance was higher for short (≤30 days) and longer (>90 days) treatment regimens than for treatment regimens lasting for 31 to 90 days (*p* = 0.014). A lower number of inpatient days during the 12 months prior to study entry was also associated with higher levels of compliance. Post-baseline variables associated with high compliance were greater life satisfaction and less work impairment during the study. There was also an association with time, indicating increased compliance from one visit to the next.

**Table 3:** **Factors associated with compliance during the study**

**Variables**	**Value**	**Odds ratio**	**95% CI**	***p*** **value (vs reference)**	***p*** **value (overall)**
Compliance at baseline	Low (<80%)	1	Reference		<0.001
	High (80% to 100%)	6.91	[5.04, 9.46]	<0.001	
Country					<0.001
Length of initial treatment	>90 days	1	Reference		0.014
	31 to 90 days	0.74	[0.54, 1.03]	0.073	
	30 days or fewer	1.42	[0.89, 2.27]	0.14	
Insight into illness at baseline	Moderate	1	Reference		<0.001
	Low/none	0.33	[0.20, 0.56]	<0.001	
	Medium	0.94	[0.65, 1.35]	0.73	
	High	2.79	[1.87, 4.18]	<0.001	
Number of days the patient used day hospital		0.99	[0.98, 0.99]		0.002
Satisfaction with life during study	Very satisfied	1	Reference		0.002
	Satisfied	0.65	[0.48, 0.88]	0.006	
	Neither satisfied nor dissatisfied	0.56	[0.37, 0.83]	0.004	
	Dissatisfied	0.39	[0.24, 0.62]	<0.001	
	Very dissatisfied	0.81	[0.39, 1.67]	0.57	
Impairment in work activities during study	Severe	1	Reference		0.007
	Moderate	0.91	[0.53, 1.57]	0.75	
	Mild	1.15	[0.69, 1.92]	0.59	
	No	1.64	[0.92, 2.92]	0.09	
Time of observation	Per visit				0.013

CI, confidence interval.

### Symptoms and relapse during the study

**Figure 3:**
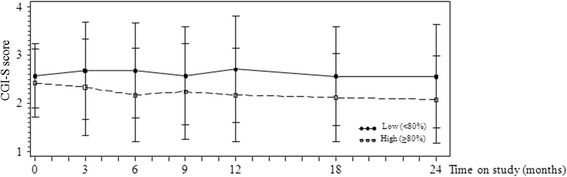
**Mean CGI-S score (±standard deviation (SD)) during the study by current compliance level.** CGI-S, Clinical Global Impression of Severity.

**Figure 4:**
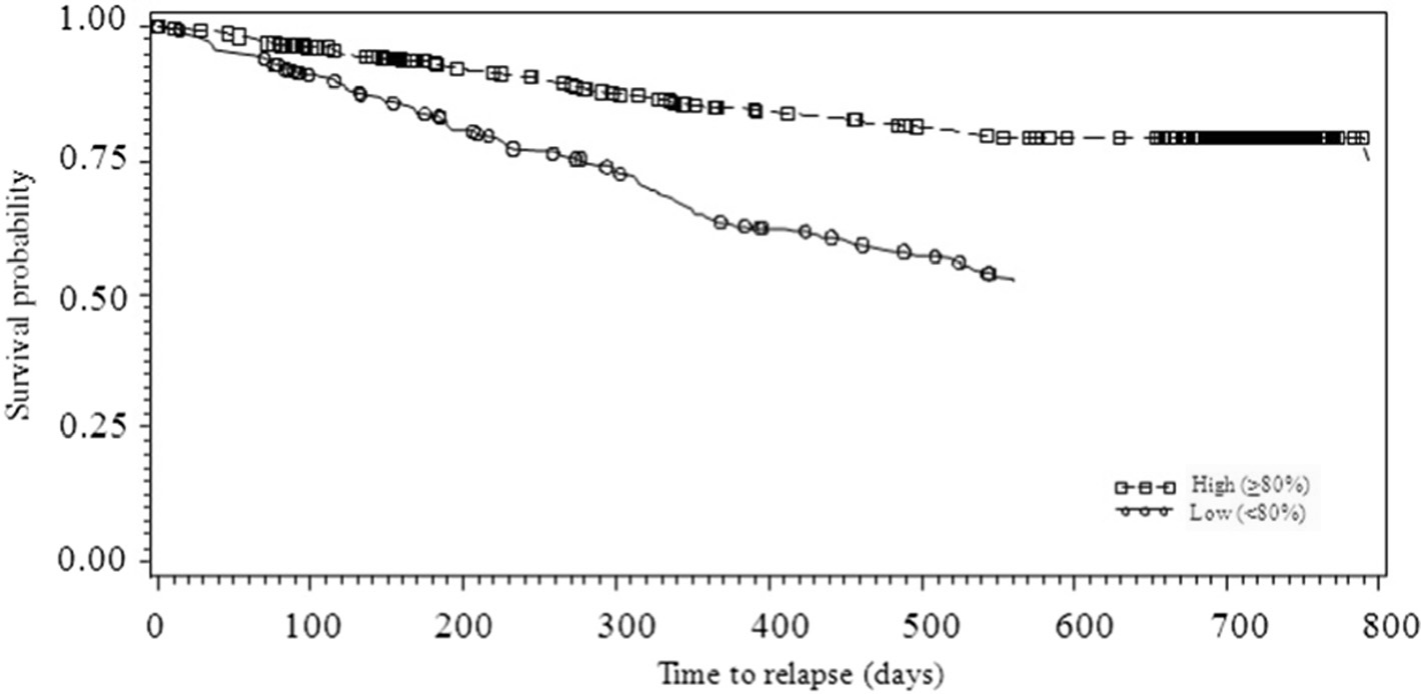
**Kaplan-Meier analysis of time to relapse.**

Figure 3 indicates that the gap between the mean CGI-S scores of patients in the HCG and LCG observed at baseline did not diminish over the course of the study. The mean CGI-S score of patients in the HCG remaining in the study decreased throughout the course of the study, whereas the CGI-S score of patients in the LCG was relatively stable (no formal comparison over time was done). Time to relapse was shorter in the LCG as illustrated in Figure 4. After adjustment for other variables, less than 80% compliance at baseline was associated with a twofold increase in the chance of relapse (Table 4), while a lower CGI-S score during the study was associated with lower odds of relapse. A difference of 1 unit (or 63% of the total possible range) in the EQ-5D overall health status score over time was associated with a fourfold decrease in the odds of relapse. Patients with an ‘excellent’ patient-physician relationship had 52% higher odds of relapse than patients with a ‘very good’ rating, and there was high variability in reports of relapse between countries (*p* < 0.001). Similarly, the time to relapse was different between countries and a difference of 1 unit in the EQ-5D overall health status score (the total EQ-5D range was −0.59 to 1) was associated with a 54% reduction in the hazard of relapse. No other baseline characteristics were associated with time to relapse.

**Table 4:** **Factors significantly associated with the absence of relapse (logistic regression results)**

**Factor**	**Value**	**OR**	**95% CI**	***p*** **value**
Compliance to treatment regimen at baseline	High vs low	2.08	[1.30, 3.33]	0.002
CGI-S over time	Per unit	0.29	[0.18, 0.46]	<0.001
Country	6 countries			<0.001
EQ-5D overall health status score over time	Per unit	4.16	[1.57, 11.04]	0.004
Patient-physician relationship	Very good	1	Reference	
	Excellent	0.48	[0.26, 0.91]	0.023
	Good	1.34	[0.80, 2.24]	0.26
	Poor	0.91	[0.35, 2.37]	0.84

CGI-S, Clinical Global Impression of Severity; CI, confidence interval; EQ-5D, European Quality of Life instrument-5 dimensions (overall score based on UK norms); OR, odds ratio.

### Association of compliance with attitude towards medication

To determine whether higher levels of compliance were associated with a more affirmative attitude towards medication as measured by the DAI-10, single DAI-10 questions and the total DAI-10 score were modeled versus compliance at each visit. Answers to questions 1 (‘Good things of the current medication outweighing the bad’ , odds ratio (OR) = 2.24 [1.64, 3.04], *p* < 0.001), 2 (‘Feeling weird on the current medication’ , OR = 0.70 [0.55, 0.88], *p* = 0.003), 3 (‘Taking medication of own choice’ , OR = 1.34 [1.07, 1.67], *p* = 0.010), and 5 (‘Feeling tired and sluggish on the current medication’ , OR = 0.82 [0.68, 0.995], *p* = 0.045) were selected as highly associated in a joint model. Similarly, the total DAI-10 score (OR = 1.08 [1.05, 1.10], *p* < 0.001) turned out to be significantly associated with compliance as a single predictor. The model fit suggests that using the total DAI-10 score as a single predictor of compliance is better than using a more complex model with the four sub-domains described earlier or the ten sub-domains of the scale.

As shown in Table 5, being older and having higher EQ-5D overall health status scores during the study, higher EQ-5D VAS scores during the study, and greater baseline insight into illness were identified as being positively associated with a more affirmative attitude towards medication. Higher CGI-S scores during the study were associated with a less affirmative attitude towards medication, and there were substantial differences in DAI-10 ratings between countries, which were adjusted for in the model.

**Table 5:** **Factors that were identified to be significantly predictive of better attitude towards medication**

**Factor**	**Estimate**	**95% CI**	***p*** **value**
Age	0.019	[0.004, 0.034]	0.012
CGI-S	−0.31	[−0.42, −0.20]	<0.001
EQ-5D (range −0.6 to 1)	1.11	[0.56, 1.66]	<0.001
EQ-5D VAS	0.02	[0.01, 0.02]	<0.001
Baseline insight into illness (vs moderate)	0		Reference
Low/none	−1.66	[−2.37, −0.94]	<0.001
Medium	−0.38	[−0.89, 0.13]	0.14
High	0.90	[0.43, 1.37]	<0.001
Country (increasing order: Taiwan, Korea, Austria, Hungary, Mexico, Romania)	0 to 3.86		<0.001

CGI-S, Clinical Global Impression of Severity; CI, confidence interval; EQ-5D, European Quality of Life instrument-5 dimensions; VAS, visual analog scale.

### Association of compliance with medical resource utilization

Based on UK estimates, a day of hospital stay costs about twice as much (£219) as day hospital or day care use (£98) or an outpatient consultation with a psychiatrist (0.5 h = £103.5). According to such a cost structure, the logistic regression model indicated that high compliance was marginally associated with lower total medical resource utilization (−£122 [−252, +9] per 3 months, *p* = 0.067), with a strong increase in costs per each unit of higher CGI-S score (+£314, *p* < 0.001), and change in costs over time (*p* = 0.001), with lower costs in the second year of the study.

### Association of compliance with quality of life

**Figure 5:**
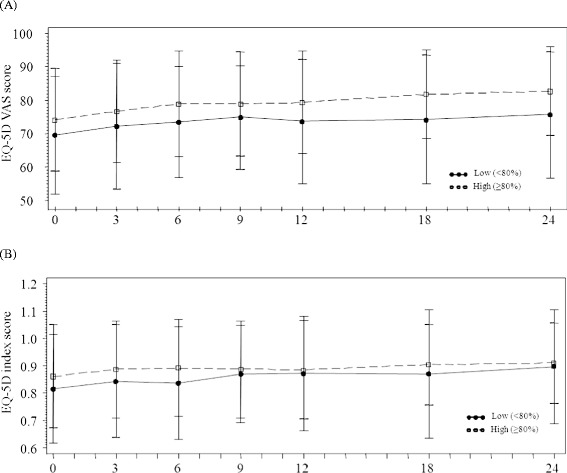
**Mean (±SD) EQ-5D VAS score (A) and mean (±SD) EQ-5D health status score (B).** EQ-5D, European Quality of Life instrument-5 dimensions; VAS, visual analog scale.

According to the GEE model, high compliance was associated with better quality of life as assessed by the EQ-5D. The increase of 1 unit (63%) in the derived index score was associated with a twofold increase in the odds of high compliance (OR = 2.2, 95% CI = [1.4, 3.5], *p* = 0.001) after adjustment for time trend. Similarly, an increase of 1% on the EQ-5D VAS scale resulted in a 1.2% increase in the odds of high compliance (95% CI = [0.7, 1.8], *p* < 0.001). Figure 5 shows the trend of the mean EQ-5D health status score and the mean EQ-5D VAS score. On both graphs, the mean EQ-5D score of patients in the HCG was higher than that of patients in the LCG throughout the study. Mean EQ-5D scores increased in both groups during the course of the study.

### Gender

Possible differences regarding compliance between male and female patients were analyzed in the present patient sample, but no statistically significant differences were found. Similarly, no correlations between gender and any of the secondary outcome parameters (e.g., DAI-10 total score, time to relapse, total medical resource utilization) were found.

### Weight gain

Collection of adverse event data was not an objective of the study since patients were receiving multiple medications in a naturalistic setting, according to the prescriptions of the treating physicians. However, patients' weight was measured at each visit. Analyses of patients while on olanzapine-containing treatment regimens indicated that weight at baseline and 24 months was similar in the HCG and LCG, with an average weight gain of approximately 2 kg being observed over the 2 years of the study, both in the HCG (mean weight at baseline 73.5 kg (standard deviation (SD) 13.8), at the 24-month visit 75.4 kg (SD 13.8)) and the LCG (mean weight at baseline 73.9 kg (SD 14.1), at the 24-month visit 75.8 kg (SD 12.9)).

## Discussion

To our knowledge, the present study is the first large, noninterventional clinical trial assessing compliance to treatment as the main outcome parameter in patients with bipolar disorder. According to our analysis and in line with everyday clinical experience, patients with ‘high’ versus ‘low’ compliance showed significant differences in a range of parameters assessed at baseline and throughout the study. Due to the observational and naturalistic study design, conclusions regarding causality between compliance and other parameters cannot be derived. The findings that high baseline compliance is a strong predictor of later compliance and the importance of the patients' attitude towards treatment suggest that compliance might be a rather stable factor in the treatment course of bipolar patients. It is also possible that the strong association between baseline and 2-year compliance may be due to both variables being secondary to one or more common factors that remained relatively stable during follow-up.

The interrelation between compliance and all other investigated variables was multifaceted: high levels of compliance at baseline and during the study were significantly associated with more favorable clinical, functional, and social outcomes in the HCG compared to the LCG, suggesting that the relationship between compliance and these factors may at least be bi-directional. While higher levels of compliance may contribute to lower CGI-S scores, better insight into illness, higher levels of social and work performance, and better quality of life, it may very well be that patients with more favorable parameters at baseline (better clinical status, less severe disease course, better disease insight, etc.) show higher levels of compliance per se and that, in this context, better compliance might be only one additional parameter contributing to a more favorable prognosis. However, even if causality between parameters cannot be determined, improving some of these variables may also have a positive influence on the other parameters (including compliance), thus allowing for other possible interventions (such as psychoeducation, improvement of patient-physician relationship, etc.) besides pharmacological treatment in the long-term management of bipolar disorder.

Overall, in our patient sample, compliance was high both at baseline and also at all follow-up visits, regardless of the use of olanzapine as monotherapy or in combination with other agents. Compliance increased among study completers, a finding that is not surprising, as ‘lost to follow-up’ and ‘patient decision’, the main reasons for dropout, could be interpreted as noncompliance to the treatment schedule. Hence, the clinical trial setting itself might be a confounder, since higher rates of compliance are likely to occur in patients willing to participate in clinical research. This suggestion is supported by the results of a study of the effectiveness of lithium in bipolar disorder, in which patients were highly compliant with their treatment regimen (Sylvia et al. 2014). To minimize this factor, a naturalistic study design was chosen to investigate compliance under conditions that mimic routine clinical care. In order to increase reliability and generalizability, a large patient population was investigated, and a follow-up period of 2 years was chosen to determine the long-term disease course of this patient sample. Furthermore, as patients from different European countries (including countries in both Western and Eastern Europe with different health-care systems), Latin America, and Asia were included in the study, results from different social and medical practices regarding compliance could be obtained.

Overall, 44% of the patients included in the present study were on olanzapine monotherapy. This surprisingly high proportion may be the result of selection bias due to the clinical trial setting. However, the use of monotherapy may also contribute to higher levels of compliance, as it is easy for patients to use. While data on combination therapy in the maintenance treatment of bipolar disorders are limited, olanzapine monotherapy has been shown in several studies to have an efficacy profile comparable to that of lithium and was therefore rated as having Level A evidence for the prophylactic treatment of bipolar patients (Grunze et al. 2004). Additionally, only stable patients who had received olanzapine treatment for at least 4 weeks before baseline and who had CGI-S scores of ≤3 were allowed to enter the trial, a constellation further contributing to the high compliance rates observed in this particular patient population. This agrees with the high rate of completers in the study, while in routine clinical practice, many patients have severe problems not only with taking their medication on a regular basis but also in attending appointments (DelBello et al. 2007; Scott and Pope 2002; Strakowski et al. 2007; Chen et al. 2013). In line with that, compliance was evaluated regarding the whole treatment regimen (medication, keeping appointments, etc.) in this trial and thus cannot be attributed completely to only one particular aspect of treatment, such as a single medication.

The rationale for including only patients who had been stabilized with olanzapine given as mono- or combination therapy was that at the start of the study (January 2005), olanzapine was the only antipsychotic approved for the treatment of bipolar disorder in the participating countries. It is possible that the inclusion of patients stabilized solely on olanzapine introduced bias into the study (Lundh et al. 2012). However, a study by Rascati et al. (2011) showed that bipolar patients taking olanzapine were 35% more likely (*p* = 0.04) to discontinue their medication than patients taking ziprasidone, and another study showed that compliance with quetiapine was marginally greater (2% to 4% greater; *p* ≤ 0.002) than that with other antipsychotics, including olanzapine (Gianfrancesco et al. 2006). Conversely, another study showed that compliance among patients with bipolar disorder was similar for the different atypical antipsychotics (Hassan et al. 2007). Thus, it is unlikely that limiting patient participation to those treated with olanzapine had a significant effect on the results of the study. Furthermore, the recruitment of such patients would have provided a consistent patient population in terms of the basic treatment received.

Several other factors may also have contributed to the high levels of compliance in our patient sample. Compliance was assessed by a subjective measurement (a physician-rated scale); however, clinicians tend to overestimate their patients' compliance rates to the prescribed treatment regimen (Osterberg and Blaschke 2005). As shown previously, there can be significant disagreement between physicians' estimates of patients' compliance and the results of pill counts or electronic monitoring (Velligan et al. 2007). In the present study, higher levels of compliance were once again shown to be associated with lower probability for relapse; therefore, our findings are in line with several other studies showing that compliance may be an important factor in the long-term management of bipolar disorder, especially with regard to relapse prevention and rehospitalization (Velligan et al. 2009; Novick et al. 2010).

In our study, compliance showed a nonlinear relationship with the duration of treatment at the first routine follow-up visit (approximately 3 months post baseline)*.* This could have been related to the fact that it is easier to be compliant in the short term and to the presence of a group of patients who are highly compliant and therefore likely to continue taking their medication for more than 3 months. In addition, there is potential for the taking of medication to become a habit in the longer term. Alternatively, it could have been due to an artifact in the study design in that patients were treated with olanzapine for at least 4 weeks before study entry, yet those who discontinued treatment by the first follow-up visit would be categorized as having a treatment duration of 31 to 90 days rather than >90 days. Further research is needed to explore the change in compliance with long-term treatment.

Despite substantial advances in pharmacological and nonpharmacological treatment possibilities, bipolar disorder often entails multiple relapses and impaired psychological functioning (Berk et al. 2010; Solomon et al. 2010). However, the extent to which modern treatments have influenced the natural course of mental disorders is not entirely clear. Providing a prognosis of the course and outcome of bipolar disorder(s) continues to be challenging, despite the variety of treatment options and multiple research efforts worldwide. Although long-term symptomatic remission does not guarantee functional recovery, it may have a favorable impact on long-term overall prognosis. The high degree of treatment resistance in patients with bipolar disorder highlights the need to develop better outcome predictors for compliance, prognosis, and treatment intervention, designed to reverse or prevent this burden of illness (Treuer and Tohen 2010). Therefore, some of our findings, such as differences in compliance rates between countries, as well as potential cultural influences and the influence of different health-care systems - factors that may influence the compliance of bipolar patients - need further investigation.

Other factors crucial for compliance, such as adverse events during treatment with antipsychotics/mood stabilizers and their impact on patients' compliance could only be evaluated to a very limited extent (and not in context with a single medication) in the present study, as combination therapy, concomitant medications, and changes in treatment regimens were allowed at all time-points during study participation. Overall weight gain was low in our olanzapine-treated patient sample, another factor that might have contributed to the high compliance rates in study completers. However, weight gain at the individual patient level showed much variability. Analyses of patients on olanzapine-containing regimens in the present study indicated less weight gain during the course of the trial compared to other studies (Lipkovich et al. 2006) and may be driven by dropouts due to weight gain. Furthermore, comorbidities and personality traits (Axelsson et al. 2009; Halimi et al. 2010; Holma et al. 2010; Jerant et al. 2011; Pappa et al. 2006; Pompili et al. 2013) - both also very likely contributing to different levels of compliance in bipolar patients - were not systematically assessed by structured interviews. Therefore, and similar to routine clinical care, these are very likely underreported in our sample and are not in accordance with published comorbidity rates in bipolar patients (especially alcohol/substance abuse, personality disorders, comorbid attention deficit hyperactivity disorder, etc.) (Correa et al. 2010; DelBello et al. 2007; Perugi et al. 2010).

One limitation of our study is the fact that no specific diagnostic instruments were used to screen for bipolar disorder. Rather, physicians were requested to establish the diagnosis according to their routine clinical approach. This is an acceptable approach given the observational nature of the study, but it is worth noting that inter-rater reliability for diagnosing bipolar I and II disorders was low in initial field trials of the fifth edition of the Diagnostic and Statistical Manual of Mental Disorders (kappa values 0.56 and 0.40, respectively) (Freedman et al. 2013). Another limitation is the fact that inter-rater reliability for the different *ad hoc* questionnaires used to measure outcomes was not evaluated because, although important for controlled clinical trials, this type of evaluation is not generally used in naturalistic studies. The reason for this is that the key goal of these studies is to investigate how such rating is performed in a ‘real world’ setting. In addition, investigators did not receive specific training for using the *ad hoc* questionnaires because they involved simple questions with a number of possible answers and were therefore very easy to administer.

Other limitations include the fact that compliance was measured by a subjective rather than objective approach (e.g., measurement of plasma olanzapine levels, pill counts, or electronic monitoring). As discussed above, physicians tend to overestimate patient compliance, which may account for the high levels of compliance observed in the study. Nevertheless, our findings are in line with several other studies showing that compliance may be an important factor in the long-term management of bipolar disorder. Additionally, although compliance results from different social and medical practices were obtained in our study due to the inclusion of patients from different countries, no statistical analyses by country were performed because such analyses would have been very limited, particularly with so much variability in the data. A final limitation is the lack of investigation of the effect of symptomatology on compliance. Patients who are more symptomatic often change medication frequently because they feel it is not effective, and this can cause them to be less compliant with their current treatment regimen. The effect of the degree of symptomatology on compliance warrants further investigation.

## Conclusions

Compliance is one of the key factors in the successful management and long-term treatment of patients with bipolar disorder. Therefore, it should be the subject of further investigation as the main outcome parameter of clinical studies. Factors that have been shown to positively influence compliance, such as the patient-physician relationship, should be given more attention in the management of bipolar patients.

## Abbreviations

CGI-S: Clinical Global Impression of Severity
CI: confidence interval
DAI-10: Drug Attitude Inventory - short version
EQ-5D: European Quality of Life instrument-5 dimensions
GEE: generalized estimating equations
HCG: high compliance group
LCG: low compliance group
OR: odds ratio
SD: standard deviation
VAS: visual analog scale

## References

[CR1] Adams J, Scott J (2000). Predicting medication adherence in severe mental disorders. Acta Psychiatr Scand.

[CR2] Awad GA (1993). Subjective response to neuroleptics in schizophrenia. Schizophr Bull.

[CR3] Axelsson M, Emilsson M, Brink E, Lundgren J, Toren K, Lötvall J (2009). Personality, adherence, asthma control and health-related quality of life in young adult asthmatics. Respir Med.

[CR4] Baker RW, Kinon BJ, Maguire GA, Liu H, Hill AL (2003). Effectiveness of rapid initial dose escalation of up to forty milligrams per day of oral olanzapine in acute agitation. J Clin Psychopharmacol.

[CR5] Berk M, Hallam K, Malhi GS, Henry L, Hasty M, Macneil C, Yucel M, Pantelis C, Murphy B, Vieta E, Dodd S, McGorry PD (2010). Evidence and implications for early intervention in bipolar disorder. J Ment Health.

[CR6] Beynon S, Soares-Weiser K, Woolacott N, Duffy S, Geddes JR (2009). Pharmacological interventions for the prevention of relapse in bipolar disorder: a systematic review of controlled trials. J Psychopharmacol.

[CR7] Chen W, Deveaugh-Geiss AM, Palmer L, Princic N, Chen YT (2013). Patterns of atypical antipsychotic therapy use in adults with bipolar I disorder in the USA. Hum Psychopharmacol.

[CR8] Colom F, Vieta E (2004). Improving the outcome of bipolar disorder through non-pharmacological strategies: the role of psychoeducation. Rev Bras Psiquiatr.

[CR9] Correa R, Akiskal H, Gilmer W, Nierenberg AA, Trivedi M, Zisook S (2010). Is unrecognized bipolar disorder a frequent contributor to apparent treatment resistant depression?. J Affect Disord.

[CR10] Cramer J, Rosenheck R (1998). Compliance with medication regimens for mental and physical disorders. Psychiatr Serv.

[CR11] Cramer J, Rosenheck R, Kirk G, Krol W, Krystal J, VA Naltrexone Study Group 425 (2003). Medication compliance feedback and monitoring in a clinical trial: predictors and outcomes. Value Health.

[CR12] DelBello M, Hanserman D, Adler CM, Fleck DE, Strakowski SM (2007). Twelve-month outcome of adolescents with bipolar disorder following first hospitalization for manic or mixed episode. Am J Psychiatry.

[CR13] Freedman R, Lewis DA, Michels R, Pine DS, Schultz SK, Tamminga CA, Gabbard GO, Gau SS, Javitt DC, Oquendo MA, Shrout PE, Vieta E, Yager J (2013). The initial field trials of DSM-5: new blooms and old thorns. Am J Psychiatry.

[CR14] Gianfrancesco FD, Rajagopalan K, Sajatovic M, Wang RH (2006). Treatment adherence among patients with bipolar or manic disorder taking atypical and typical antipsychotics. J Clin Psychiatry.

[CR15] Gonzalez-Pinto A, Mosquera F, Alonso M, López P, Ramírez F, Vieta E, Baldessarini RJ (2006). Suicidal risk in bipolar I disorder patients and adherence to long-term lithium treatment. Bipolar Disord.

[CR16] Goodwin GM, for the Consensus Group of the British Association for Psychopharmacology (2003). Evidence-based guidelines for treating bipolar disorder: recommendations from the British Association for Psychopharmacology. J Psychopharmacol.

[CR17] Goodwin GM, for the Consensus Group of the British Association for Psychopharmacology (2009). Evidence-based guidelines for treating bipolar disorder: revised second edition—recommendations from the British Association for Psychopharmacology. J Psychopharmacol.

[CR18] Greenberg RN (1984). Overview of patient compliance with medication dosing: a literature review. Clin Ther.

[CR19] Grunze H, Kasper S, Goodwin G, Bowden C, Möller HJ, WFSBP Task Force on Treatment Guidelines for Bipolar Disorders (2004). The World Federation of Societies of Biological Psychiatry (WFSBP) guidelines for the biological treatment of bipolar disorders, part III: maintenance treatment. Word J Biol Psychiatry.

[CR20] Grunze H, Vieta E, Goodwin GM, Bowden C, Licht RW, Möller HJ, Kasper S, WFSBP Task Force on Treatment Guidelines for Bipolar Disorders (2009). The World Federation of Societies of Biological Psychiatry (WFSBP) guidelines for the biological treatment of bipolar disorders: update 2009 on the treatment of acute mania. World J Biol Psychiatry.

[CR21] Grunze H, Vieta E, Goodwin GM, Bowden C, Licht RW, Möller HJ, Kasper S, WFSBP Task Force on Treatment Guidelines for Bipolar Disorders (2010). The World Federation of Societies of Biological Psychiatry (WFSBP) guidelines for the biological treatment of bipolar disorders: update 2010 on the treatment of acute bipolar depression. World J Biol Psychiatry.

[CR22] Guy W (1976). Clinical global impressions. ECDEU assessment manual for psychopharmacology, revised.

[CR23] Halimi L, Pry R, Pithon G, Godard P, Varrin M, Chanez P (2010). Severe asthma and adherence to peak flow monitoring: longitudinal assessment of psychological aspects. J Psychosom Res.

[CR24] Hassan M, Madhavan SS, Kalsekar ID, Makela EH, Rajagopalan K, Islam S, Kavookjian J, Miller LA (2007). Comparing adherence to and persistence with antipsychotic therapy among patients with bipolar disorder. Ann Pharmacother.

[CR25] Haynes RB, McDonald HP, Garg AX (2002). Helping patients follow prescribed treatment: clinical applications. JAMA.

[CR26] Hirschfield RMA (2014). Guideline watch: practice guideline for the treatment of patients with bipolar disorder.

[CR27] Holma IA, Holma KM, Melartin TK, Isometsä ET (2010). Treatment attitudes and adherence of psychiatric patients with major depressive disorder: a five-year prospective study. J Affect Disord.

[CR28] Jackevicius CA, Mamdani M, Tu JV (2002). Adherence with statin therapy in elderly patients with and without acute coronary syndromes. JAMA.

[CR29] Jerant A, Chapman B, Duberstein P, Robbins J, Franks P (2011). Personality and medication non-adherence among older adults enrolled in a six-year trial. Br J Health Psychol.

[CR30] Judd LL, Akiskal HS (2003). The prevalence and disability of bipolar spectrum disorders in the US population: re-analysis of the ECA database taking into account subthreshold cases. J Affect Disord.

[CR31] Li C, Chen C, Qiu B, Yang G (2014). A 2-year follow-up study of discharged psychiatric patients with bipolar disorder. Psychiatry Res.

[CR32] Lipkovich I, Citrome L, Perlis R, Deberdt W, Houston JP, Ahl J, Hardy T (2006). Early Predictors of substantial weight gain in bipolar patients treated with olanzapine. J Clin Psychopharmacol.

[CR33] Lundh A, Sismondo S, Lexchin J, Busuioc OA, Bero L (2012). Industry sponsorship and research outcome. Cochrane Database Syst Rev.

[CR34] Murray CJ, Lopez AD (1996). Evidence-based health policy-lessons from the Global Burden of Disease Study. Science.

[CR35] Novick D, Haro JM, Suarez D, Perez V, Dittmann RW, Haddad PM (2010). Predictors and clinical consequences of non-adherence with antipsychotic medication in the outpatient treatment of schizophrenia. Psychiatry Res.

[CR36] Osterberg L, Blaschke T (2005). Adherence to medication. N Engl J Med.

[CR37] Pappa C, Hyphantis T, Pappa S, Aspiotis M, Stefaniotou M, Kitsos G, Psilas K, Mavreas V (2006). Psychiatric manifestations and personality traits associated with glaucoma treatment. J Psychsom Res.

[CR38] Perugi G, Frare F, Toni C, Tusini G, Vannucchi G, Akiskal HS (2010). Adjunctive valproate in panic disorder patients with comorbid bipolar disorder or otherwise resistant to standard antidepressants: a 3-year “open” follow-up study. Eur Arch Psychiatry Clin Neurosci.

[CR39] Pompili M, Venturini P, Palermo M, Stefani H, Seretti ME, Lamis DA, Serafini G, Amore M, Girardi P (2013). Mood disorders medications: predictors of nonadherence - review of the current literature. Expert Rev Neurother.

[CR40] Rascati KL, Richards KM, Ott CA, Goddard AW, Stafkey-Mailey D, Alvir J, Sanders KN, Mychaskiw M (2011). Adherence, persistence of use, and costs associated with second-generation antipsychotics for bipolar disorder. Psychiatr Serv.

[CR41] Regeer EJ, ten Have M, Rosso ML, Hakkaart-van Roijen L, Vollebergh W, Nolen WA (2004). Prevalence of bipolar disorder in the general population: a reappraisal study of the Netherlands Mental Health Survey and Incidence Study. Acta Psychiatr Scand.

[CR42] Scott J, Pope M (2002). Non-adherence with mood stabilizers: prevalence and predictors. J Clin Psychiatry.

[CR43] Sharma PS, Kongasseri S, Praharaj SK (2014). Outcome of mood stabilizer discontinuation in bipolar disorder after 5 years of euthymia. J Clin Psychopharmacol.

[CR44] Simhandl C, König B, Amann BL (2014). A prospective 4-year naturalistic follow-up of treatment and outcome of 300 bipolar I and II patients. J Clin Psychiatry.

[CR45] Solomon DA, Leon AC, Coryell WH, Endicott J, Li C, Fiedorowicz JG, Boyken L, Keller MB (2010). Longitudinal course of bipolar I disorder: duration of mood episodes. Arch Gen Psychiatry.

[CR46] Strakowski SM, Tsai SY, DelBello MP, Chen CC, Fleck DE, Adler CM, Arndt S, Amicone J (2007). Outcome following a first manic episode: cross-national U.S. and Taiwan comparison. Bipolar Disord.

[CR47] Sun SX, Liu GG, Christensen DB, Fu AZ (2007). Review and analysis of hospitalization costs associated with antipsychotic nonadherence in the treatment of schizophrenia in the United States. Curr Med Res Opin.

[CR48] Sylvia LG, Reilly-Harrington NA, Leon AC, Kansky CI, Calabrese JR, Bowden CL, Ketter TA, Friedman ES, Iosifescu DV, Thase ME, Ostacher MJ, Keyes M, Rabideau D, Nierenberg AA (2014). Medication adherence in a comparative effectiveness trial for bipolar disorder. Acta Psychiatr Scand.

[CR49] The EuroQol Group (1990). EuroQol–a new facility for the measurement of health-related quality of life. Health Policy.

[CR50] Tohen M, Sanger TM, McElroy SL, Tollefson GD, Chengappa KN, Daniel DG, Petty F, Centorrino F, Wang R, Grundy SL, Greaney MG, Jacobs TG, David SR, Toma V (1999). Olanzapine versus placebo in the treatment of acute mania. Olanzapine HGEH Study Group. Am J Psychiatry.

[CR51] Tohen M, Chengappa KN, Suppes T, Zarate CA, Calabrese JR, Bowden CL, Sachs GS, Kupfer DJ, Baker RW, Risser RC, Keeter EL, Feldman PD, Tollefson GD, Breier A (2002). Efficacy of olanzapine in combination with valproate or lithium in the treatment of mania in patients partially nonresponsive to valproate or lithium monotherapy. Arch Gen Psychiatry.

[CR52] Tohen M, Vieta E, Calabrese J, Ketter TA, Sachs G, Bowden C, Mitchell PB, Centorrino F, Risser R, Baker RW, Evans AR, Beymer K, Dube S, Tollefson GD, Breier A (2003). Efficacy of olanzapine and olanzapine-fluoxetine combination in the treatment of bipolar I depression. Arch Gen Psychiatry. 60:1079-88. Erratum in. Arch Gen Psychiatry 2004.

[CR53] Tohen M, Greil W, Calabrese JR, Sachs GS, Yatham LN, Oerlinghausen BM, Koukopoulos A, Cassano GB, Grunze H, Licht RW, Dell'Osso L, Evans AR, Risser R, Baker RW, Crane H, Dossenbach MR, Bowden CL (2005). Olanzapine versus lithium in the maintenance treatment of bipolar disorder: a 12-month, randomized, double-blind, controlled clinical trial. Am J Psychiatry.

[CR54] Tohen M, Calabrese JR, Sachs GS, Banov MD, Detke HC, Risser R, Baker RW, Chou JC, Bowden CL (2006). Randomized, placebo-controlled trial of olanzapine as maintenance therapy in patients with bipolar I disorder responding to acute treatment with olanzapine. Am J Psychiatry.

[CR55] Treuer T, Tohen M (2010). Predicting the course and outcome of bipolar disorder: a review. Eur Psychiatry.

[CR56] Velligan DI, Wang M, Diamond P, Glahn DC, Castillo D, Bendle S, Lam YW, Ereshefsky L, Miller AL (2007). Relationships among subjective and objective measures of adherence to oral antipsychotic medications. Psychiatr Serv.

[CR57] Velligan DI, Weiden PJ, Sajatovic M, Scott J, Carpenter D, Ross R, Docherty JP (2009). The expert consensus guideline series: adherence problems in patients with serious and persistent mental illness. J Clin Psychiatry.

[CR58] Yatham LN, Kennedy SH, O'Donovan C, Parikh SV, MacQueen G, McIntyre RS, Sharma V, Beaulieu S, Guidelines Group, CANMAT (2006). Canadian Network for Mood and Anxiety Treatments (CANMAT) guidelines for the management of patients with bipolar disorder: update 2007. Bipolar Disord.

[CR59] Yatham LN, Kennedy SH, Parikh SV, Schaffer A, Beaulieu S, Alda M, O'Donovan C, MacQueen G, McIntyre RS, Sharma V, Ravindran A, Young LT, Milev R, Bond DJ, Frey BN, Goldstein BI, Lafer B, Birmaher B, Ha K, Nolen WA, Berk M (2013). Canadian Network for Mood and Anxiety Treatments (CANMAT) and International Society for Bipolar Disorders (ISBD) collaborative update of CANMAT guidelines for the management of patients with bipolar disorder: update 2013. Bipolar Disord.

[CR60] Zygmunt A, Olfson M, Boyer CA, Mechanic D (2002). Interventions to improve medication adherence in schizophrenia. Am J Psychiatry.

